# Mixture Concentration-Response Modeling Reveals Antagonistic Effects of Estradiol and Genistein in Combination on Brain Aromatase Gene (*cyp19a1b*) in Zebrafish

**DOI:** 10.3390/ijms19041047

**Published:** 2018-04-01

**Authors:** Nathalie Hinfray, Cleo Tebby, Benjamin Piccini, Gaelle Bourgine, Sélim Aït-Aïssa, Jean-Marc Porcher, Farzad Pakdel, François Brion

**Affiliations:** 1 Institut National de l’Environnement Industriel et des Risques (INERIS), Unité d’Ecotoxicologie in vitro et in vivo, 60550 Verneuil-en-Halatte, France; benjamin.piccini@ineris.fr (B.P.); selim.ait-aissa@ineris.fr (S.A.-A.); jean-marc.porcher@ineris.fr (J.-M.P.); francois.brion@ineris.fr (F.B.); 2 Institut National de l’Environnement Industriel et des Risques (INERIS), Unité de modélisation pour la Toxicologie et l’Ecotoxicologie, 60550 Verneuil-en-Halatte, France; cleo.tebby@ineris.fr; 3 Institut de Recherche en Santé, Environnement et Travail (IRSET), Inserm U1085, Unité Transcription, Environnement et Cancer (TREC), Université de Rennes 1, 35000 Rennes, France; gaelle.bourgine@univ-rennes1.fr (G.B.); farzad.pakdel@univ-rennes1.fr (F.P.)

**Keywords:** estradiol, genistein, mixture, aromatase B, transgenic zebrafish, U251-MG

## Abstract

Comprehension of compound interactions in mixtures is of increasing interest to scientists, especially from a perspective of mixture risk assessment. However, most of conducted studies have been dedicated to the effects on gonads, while only few of them were. interested in the effects on the central nervous system which is a known target for estrogenic compounds. In the present study, the effects of estradiol (E2), a natural estrogen, and genistein (GEN), a phyto-estrogen, on the brain ER-regulated *cyp19a1b* gene in radial glial cells were investigated alone and in mixtures. For that, zebrafish-specific in vitro and in vivo bioassays were used. In U251-MG transactivation assays, E2 and GEN produced antagonistic effects at low mixture concentrations. In the *cyp19a1b*-GFP transgenic zebrafish, this antagonism was observed at all ratios and all concentrations of mixtures, confirming the in vitro effects. In the present study, we confirm (i) that our in vitro and in vivo biological models are valuable complementary tools to assess the estrogenic potency of chemicals both alone and in mixtures; (ii) the usefulness of the ray design approach combined with the concentration-addition modeling to highlight interactions between mixture components.

## 1. Introduction

Compounds able to interact with the estrogen receptors (ER) have been extensively studied over the years due to the threat they represent to aquatic species and particularly to fish reproduction and development [1,2]. However, in the aquatic environment, fish are often exposed not only to estrogenic chemicals alone but rather to mixtures, highlighting the constant need to understand compound interactions in mixtures and to develop new assays and approaches to this end.

Several studies have evaluated the combined effects of estrogenic compounds on aquatic organisms [3,4,5,6,7,8,9,10,11,12], generally concluding on an additive effect in mixtures both in vivo and in vitro. However, most in vivo studies addressed the effects of mixtures of estrogenic compounds on gonads and other peripheral organs, while only few of them studied effects on the central nervous system despite increasing evidences that estrogenic compounds interfere in neuroendocrine regulations.

In fish, the *cyp19a1b* gene encodes the brain aromatase. In zebrafish, *cyp19a1b* is expressed in radial glial cells of the brain which are crucial neuronal progenitors [13,14]. Estrogenic compounds are known to highly up-regulate *cyp19a1b* gene expression by an ER-dependent mechanism [3,15]. By using zebrafish-specific in vitro and in vivo bioassays based on the *cyp19a1b* gene coupled to a complete modeling approach, we recently demonstrated additive effects of ethynilestradiol and levonorgestrel, a pro-estrogenic compound, on *cyp19a1b* gene in glial cells [12].

In this context, the present study aimed to investigate the effects of single and combined exposure to two estrogens with different estrogenic potencies, on the expression of the zebrafish *cyp19a1b* gene. For this purpose, two in vitro and in vivo bioassays based on the zebrafish *cyp19a1b* gene were used: (i) an ER-negative human glial cell culture (U251-MG) co-transfected with two different zebrafish ER subtypes (zfERα and zfERβ2) and a luciferase gene under the control of the zebrafish *cyp19a1b* promoter [16] and (ii) a transgenic zebrafish (*cyp19a1b*-GFP) line expressing GFP under the control of the zebrafish *cyp19a1b* promoter which is used to evaluate estrogenicity of chemicals at the embryo-larval stage [3,6]. Mixtures assessed were composed of a natural potent estrogen, i.e., 17β-estradiol (E2), and a weaker estrogen, i.e., genistein (GEN), a major phytoestrogen of the isoflavone class. GEN, a human ERβ selective activator, was shown to be estrogenic in diverse in vitro and in vivo assays (for review see [17]), including in the U251-MG and in transgenic *cyp19a1b*-GFP zebrafish bioassays [3,16]. In the end, this comparative in vitro and in vivo approach was used to (i) determine the potential interactions between E2 and GEN in mixtures and (ii) evaluate the complementarity of the in vitro and in vivo models used in these experiments. The concentration-addition (CA) model for mixtures was used as the reference no-interaction model. Deviations from the CA model were quantified in terms of antagonism or synergism by using Jonker et al. interaction models [18] and their statistical significance was tested. Thereby, the present study reports antagonistic effects of E2 and GEN in mixtures on the expression of the ER-regulated gene *cyp19a1b* in a glial cell context.

## 2. Results

### 2.1. In Vitro Effects of Single Test Compounds

The effects of E2 and GEN alone were assessed in U251-MG glial cells co-transfected with zfERs and the zf-*cyp19a1b* promoter-luciferase reporter. One concentration-response experiment was carried out for each compound and each ER with three replicate wells for each condition. Luciferase activity was induced by E2 treatment in a concentration-dependent manner (Supplementary Figure S1). The EC50s (median effective concentration) were estimated at 1.86 × 10^−10^ M for ERα and 1.18 × 10^−10^ M for ERβ2. EC50s for GEN were estimated at 5.05 × 10^−8^ M for ERα and 2.96 × 10^−9^ M for ERβ2.

### 2.2. In Vitro Effects of Binary Mixtures of E2 and GEN

To confirm EC50s previously obtained in single test compound experiments, EC50s for E2 and GEN were also estimated from mixture experiments. EC50s for E2 were 4.29 × 10^−11^ and 1.38 × 10^−11^ M for U251-MG glial cells transfected with ERα and ERβ2 respectively. For GEN, EC50s were 1.68 × 10^−8^ and 4.78 × 10^−9^ M for U251-MG glial cells transfected with ERα and ERβ2 respectively. The U251-MG cells were slightly more sensitive to estradiol in the mixture experiments (EC50s were 2 to ten-fold lower than in the single compound experiments), but the relative potency on ERα was relatively unchanged (1.5 factor) and the relative potency on ERβ2 changed over ten-fold, so that in the mixture experiment the relative potencies were the same on ERα and ERβ2.

**Figure 1:**
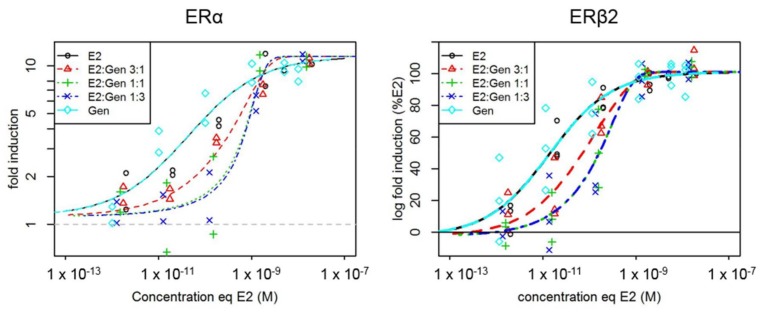
Concentration-response curves of luciferase activity in U251-MG cells transfected with ERα or ERβ2 after exposure to estradiol (E2) and genistein (GEN) alone or in combinations (three different ratios of substances). These data originated from 2 (ERα) or 3 (ERβ2) independent experiments. All the data were modeled by the dose-level dependent interaction model (DL). Each point represents the mean of triplicated wells. E2 and GEN concentration-response curves are superimposed because the concentration is expressed in E2-equivalents which have been calculated with the EC50s from these curves.

**Figure 2:**
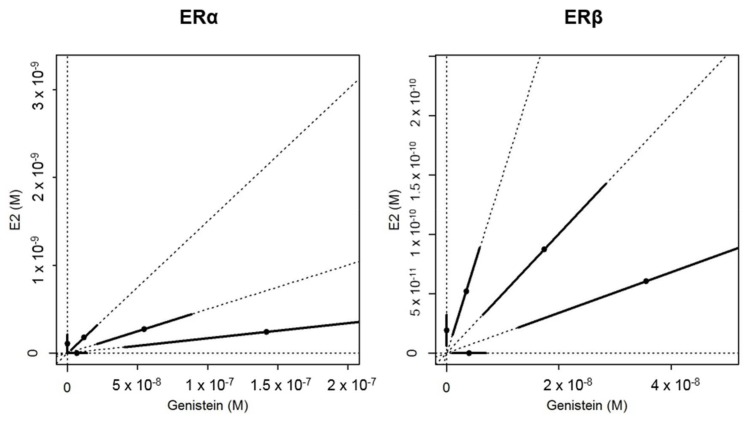
Illustration of the EC50 for each ray of the estradiol (E2) + genistein mixtures (EC50s isobologram). The points represent the EC50 and the bars represent the 95% confidence interval. These data originated from the in vitro assays with U251-MG cells transfected with the promoter of the zebrafish *cyp19a1b* gene coupled to the luciferase reporter gene and the zebrafish ERs (ERα or ERβ2). The isobole is the line formed when EC50s of each ray are joined. A straight isobole would indicate additivity. The deviation of the isobole to the right indicates an antagonism.

In vitro, a concentration-dependent induction of luciferase activity was measured with the three different mixture ratios of E2 and GEN in U251-MG cells transfected both with ERα and ERβ2 (Figure 1). The mixture model that displayed the best fit to the in vitro data was the dose-level dependent interaction model (DL) with identical slopes for the single compounds [18]. This model showed a significant improvement of the goodness of fit as compared to the CA model (approximate *F*-tests: *p* = 7.9 × 10^−4^ for ERα and *p* = 8.4 × 10^−3^ for ERβ2). Interaction parameters of the DL model (α = 19.3 and β = 0.0248 for ERα, α = 12.9 and β = 0.0158 for ERβ2) indicated strong antagonism at low concentrations between E2 and GEN in mixtures for both estrogen receptors. This antagonism was also highlighted by the deviation of the EC50s isoboles observed in the two experiments (Figure 2).

### 2.3. In Vivo Effects of Single Test Compounds

No effect due to chemical exposure was observed on lethality or time to hatch during any of the in vivo studies at the concentrations used in the experiments reported here.

**Figure 3:**
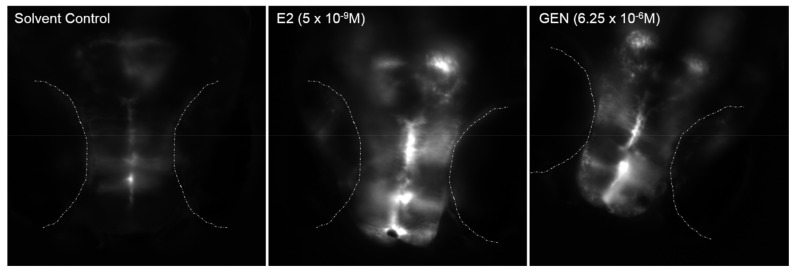
In vivo imaging of transgenic *cyp19a1b*-GFP zebrafish embryos (4-dpf old) exposed to solvent (DMSO), estradiol (E2) or genistein (GEN) for 96 h. Dorsal view of the brain showing GFP induction in the radial glial cells. For each chemical, the concentration used is indicated. Dotted lines delimit the eyes.

A 96-h exposure to E2 led to a concentration-dependent induction of GFP expression in transgenic *cyp19a1b*-GFP zebrafish (Supplementary Figure S2), with an EC50 of 2.25 × 10^−10^ M (4 independent experiments with 8–19 transgenic zebrafish per condition). GFP expression is detected in radial glial cells of the brain (Figure 3). A concentration-dependent induction of GFP expression in radial glial cells of the brain after exposure to GEN was measured with an EC50 of 1.45 × 10^−6^ M (Supplementary Figure S2) (2 experiments with 8–19 transgenic zebrafish per condition).

### 2.4. In Vivo Effects of Binary Mixtures of E2 and GEN

In the two independent mixture experiments with transgenic *cyp19a1b*-GFP zebrafish, estimated EC50s for single compounds were 2.69 × 10^−10^ M for E2 and 7.48 × 10^−7^ M for GEN. The relative potency based on these EC50s is of the same order of magnitude as in the single compound experiments.

**Figure 4:**
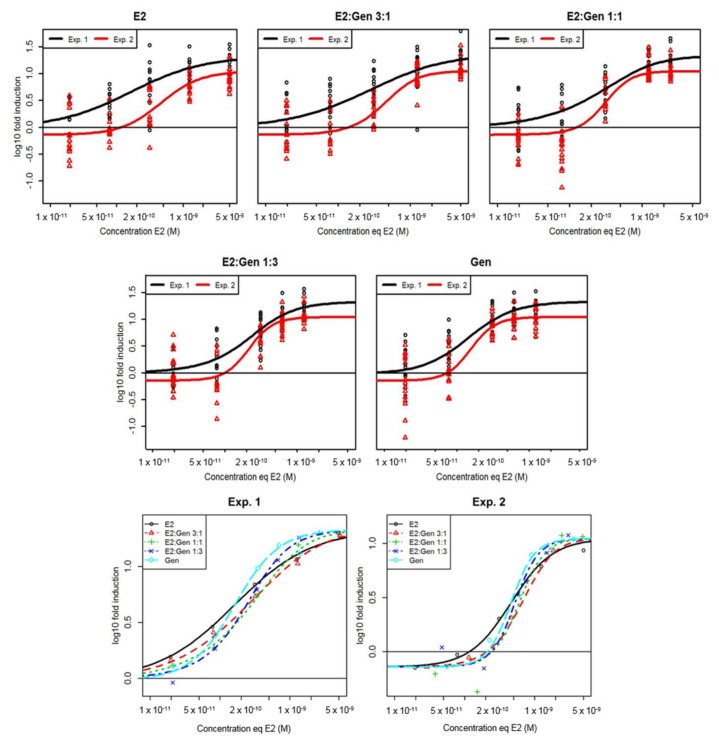
Concentration-response curves of GFP in *cyp19a1b*-GFP transgenic zebrafish after exposure to estradiol (E2) and genistein (GEN) alone or in combinations (3 different mixture ratios). These data originated from two independent experiments (Exp. 1 and Exp. 2). All the data were modeled by the simple interaction (SA) model. In the first five graphics, each point represents one measure of GFP in one transgenic fish brain (*n* = 8–19 fish per condition). In the last two graphics (bottom) which gather all the concentration-response curves, the points represent the means of the GFP experimentally measured for each experiment.

**Figure 5:**
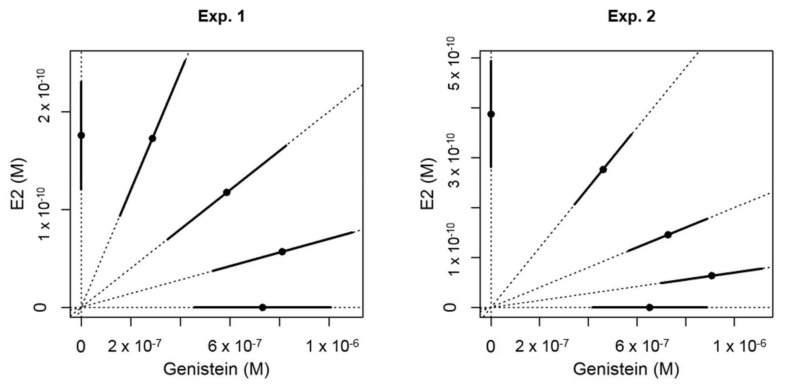
Illustration of the EC50 for each ray of the estradiol (E2) + genistein mixtures. The points represent the EC50 and the bars represent the standard error. These data originated from two independent exposure experiments with the *cyp19a1b*-GFP transgenic zebrafish line (described in Figure 4). The isobole is the line formed when EC50s of each ray are joined. A straight isobole would indicate additivity. The deviation of the isobole to the right indicates an antagonism.

In transgenic *cyp19a1b*-GFP zebrafish, the mixtures of E2 and GEN induced GFP expression in radial glial cells in a concentration-dependent manner (Figure 4). Observed responses were compared to the concentration-response surfaces modeled with the CA model with different slopes for each compound to improve the model goodness of fit [19] and were in good agreement in the two independent experiments (lack-of-fit *F*-test compared to the analysis of variance model: *p* = 0.755 (experiment 1) and *p* = 0.195 (experiment 2)) indicating that the CA model was not rejected. Interactions were then added to the CA model [18]. The simple interaction model (SA) showed a significant improvement of the adjustment quality of the model (approximate *F*-tests: *p* = 0.00325 for experiment 1 and *p* = 0.0276 for experiment 2) with interaction parameters of 2.19 (experiment 1) and 1.54 (experiment 2), indicating antagonism between E2 and GEN. This antagonism was also highlighted by the deviation of the isoboles observed in the two experiments (Figure 5). The simple antagonism model was the one finally accepted as none of the two-parameters models (dose-ratio (DR) and DL) improved goodness of fit compared to the SA model.

## 3. Discussion

In the present study, natural estrogen (E2) and phytoestrogen (GEN) were shown to be potent inducers of the ER-regulated *cyp19a1b* gene both in vitro and in vivo. In the *cyp19a1b*-GFP transgenic zebrafish line, E2 up-regulated GFP expression in radial glial cells of the brain. In a previous study, a similar EC50 was reported for the same in vivo biological model [3]. As previously reported for EE2, EC50 reported for E2 in an ERE-GFP transgenic zebrafish line [20] is over ten times higher than that calculated in the *cyp19a1b*-GFP transgenic zebrafish line, confirming the high sensitivity of this biological in vivo model to (xeno-)estrogens. In U251-MG cells transfected with ERα or ERβ2, calculated EC50s are equivalent to those reported for the *cyp19a1b*-GFP transgenic zebrafish line and for transactivation assays using human embryonic kidney (293HEK) cells [20] and zebrafish liver cells (ZELH) [21] transfected with zfERs. However, 293HEK cells displayed a greater sensitivity to E2 when transfected with human ERs than with zfERs, supporting the relevance of using fish models to evaluate potential effects of compounds on fish. Nevertheless, our results confirm that the two biological models used in the present study are sensitive and reliable tools for the study of estrogenic potency of chemicals.

GEN is a phytoestrogen with several effects on fish reproduction and development (for a review see [22]), and is known to have a lower estrogenic potency than E2. In U251-MG cells, EC50s calculated for GEN were 270 (ERα) and 25 (ERβ2) times higher than those calculated for E2 (based on single compound experiments), confirming the higher estrogenic potency of E2. In other human cell lines (MELN with endogenous human ERs, HELN transfected with human ERs), EC50 reported for GEN were in the same range as in our U251-MG assays [23,24], while in fish cell lines transfected with fish ERs (PELN, PRTH, ZELH), EC50s were 10–100 times higher suggesting a lower sensitivity of these biological models to estrogens [21,25]. Interestingly, in our U251-MG cell model, GEN is a selective ERβ modulator as previously reported in humans [23]. Furthermore, while for E2, the sensitivity of *cyp19a1b* gene expression was the same in both *cyp19a1b*-GFP transgenic zebrafish and U251-MG cells; for GEN, the transgenic zebrafish line was less sensitive than the U251-MG cells. These differences might be related to the presence in the entire organism (transgenic zebrafish) of metabolic capacities, including phase I and II biotransformation and efflux transporter proteins. Such metabolic processes might help reducing GEN availability for the ERs in radial glial cells, leading to higher EC50s in vivo. Although high GEN concentrations are necessary to induce *cyp19a1b* expression in the *cyp19a1b*-GFP transgenic zebrafish assay, response to GEN seems to be more sensitive compared to another in vivo short-term (48 h) zebrafish embryo assay using morphological defects as endpoints (edema, head and tail malformation, reduced spontaneous movement and blood circulation) (EC50 for GEN of 427 µg/L in *cyp19a1b*-GFP transgenic zebrafish vs. 2.8 mg/L) [26]. Moreover, it is of interest to note that, in the *cyp19a1b*-GFP transgenic zebrafish assay, the effects were observed in the central nervous system, i.e., radial glial cells that are progenitor cells of the brain, in early-life stage fish exposed for very short periods.

In the present study, the effects of mixtures of E2 and GEN on the expression of zebrafish *cyp19a1b* gene were also addressed both in vitro and in vivo. By this approach, antagonistic effects of E2 and GEN in mixtures on *cyp19a1b* gene expression in a glial cell context were highlighted. In vivo in transgenic *cyp19a1b*-GFP zebrafish, these antagonistic effects were observed at all concentrations and ratios of mixtures while in vitro in U251-MG cells, the antagonism was underlined at low concentrations for both estrogen receptors. Even if mixtures of estrogenic compounds usually lead to additive effects, some deviations from the CA model (antagonisms/synergisms) have already been reported both in vitro [8,27,28] and in vivo [5,29]. As regards E2 and GEN mixtures, only additive effects were observed in MCF-7 human breast cancer cells on the ER-dependent proliferation process [30,31]. However, in MCF-7 cells transfected with an ER-reporter gene transactivation system, E2 and GEN exerted an antagonistic interaction at low concentrations of mixtures [32] just like the effects measured in our U251-MG cells model. To our knowledge, in the literature, no antagonism of E2 and GEN in mixtures was reported for in vivo experiments, however, the results obtained in the present study in the *cyp19a1b*-GFP transgenic zebrafish usefully confirmed the antagonistic effects of these compounds in a glial cell context. The origin of the antagonistic interaction observed both in vitro and in vivo is not known but it may rely on the differing abilities of E2 and GEN to recruit ERs and/or coregulators as previously showed in human cell lines [33,34]. Overall, these results clearly demonstrate the importance of our two biological models for the study of mixtures since they both highlighted the antagonistic effects of E2 and GEN in mixtures on the *cyp19a1b* gene expression.

## 4. Materials and Methods

### 4.1. Compounds

E2 (purity ≥ 98%, CAS number: 50-28-2; ref E8875) and GEN (purity ≥ 98%; CAS number: 446-72-0; ref G6649) were purchased from Sigma-Aldrich (Saint Quentin Fallavier, France). 

### 4.2. Zebrafish Maintenance and Breeding

The *cyp19a1b-*GFP transgenic zebrafish [35] were raised in our laboratory facility at INERIS (Institut National de l’Environnement Industriel et des Risques, Verneuil-en-Halatte, France). They were maintained in a recirculation system (Zebtec, Tecniplast, Buguggiate, Italy) filled with 3.5 L aquaria. They were kept on a 14 h light:10 h dark cycle at a temperature of 27.0 ± 2.0 °C. For reproductions, 2 males and 1 female adult fish were gathered in each aquarium. Fertilized eggs were harvested and disinfected for 5 min in water supplemented with 0.1% of commercial bleach (2.6% of sodium hypochlorite).

### 4.3. Zebrafish Exposure to Estrogenic Compounds

Fertilized *cyp19a1b*-GFP transgenic zebrafish eggs were exposed to chemicals (alone or in mixtures) or to solvent control (DMSO, 0.02% *v*/*v*) according to [12]. Each experimental condition contained 20 embryos in 100 mL of water. Embryos were exposed for 96 h between 0 and 4 days post fertilization (dpf) without water renewal. At the end of the exposure period, 4-dpf old zebrafish were processed for fluorescence measurement by image analysis. Only *cyp19a1b*-GFP transgenic zebrafish larvae were photographed at the end of the experiment. Each experiment was conducted twice independently. All experimentations were performed in accordance with the European directive 2010/63/EU for animal experimentation.

### 4.4. In Vivo Imaging

In vivo fluorescence imaging was performed according to [3]. Each live *cyp19a1b*-GFP transgenic embryo was photographed once in dorsal view using a Zeiss AxioImager Z1 fluorescence microscope equipped with an AxioCam Mrm camera (Zeiss GmbH, Göttingen, Germany). The same exposure conditions were used to acquire each photograph (X10 objective, 134 ms of fluorescent light exposure, maximal light intensity). Fluorescence quantification was performed using Image J software (available online: http://rsb.info.nih.gov/ij/). For each picture, the integrated density (IntDen) was measured, i.e., the sum of the gray-values of all the pixels within the region of interest. All gray-values of 300 or less were considered as background values.

### 4.5. U251-MG Cell Bioassay

The U251-MG (ECACC, human astrocyte) are ER-negative glial cell line and were maintained at 37 °C in 5% CO_2_ atmosphere in phenol red–free Dulbecco’s Modified Eagle’s Medium (DMEM-F12, Sigma-Aldrich, St. Louis, MO, USA) supplemented with 8% fetal calf serum (FCS), 2 mM l-glutamine, 20 U/mL penicillin, 20 μg/mL streptomycin and 50 ng/mL amphotericin B.

One day before the transfection, cells were scraped, washed and seeded at 2 × 10^4^ cells/mL in 24-well plates in fresh medium containing 8% FCS. Transfection and luciferase assays were performed as previously described [16]. Briefly, after 24 h, the medium was replaced with fresh phenol red-free DMEM containing 2% charcoal/dextran FCS. Cells were transfected with plasmid-DNA using JetPEI^TM^ reagent, as indicated by the manufacturer (Polyplus-transfection, Illkirch, France). The DNA templates for each well correspond to 25 ng of zfER expression plasmid [36], 150 ng of the zebrafish *cyp19a1b* promoter linked to the luciferase reporter plasmid [37] and 25 ng of the internal β-galactosidase control vector (CMV-βgal). After one night, medium was replaced with fresh DMEM-F12 containing 2% charcoal/dextran FCS and cells were exposed to vehicle (DMSO, 0.1% *v*/*v*) and various concentrations of the test compounds. After 36 h, the luciferase activities were determined using the luciferase assay system (Promega, Charbonnières-les-Bains, France) and the β-galactosidase activity was used to normalize transfection efficiency in all experiments. Each experiment was performed at least in triplicate and the results were expressed as fold induction relative to the vehicle.

### 4.6. Data Normalization

In the in vivo assay with *cyp19a1b*-GFP transgenic zebrafish, induction of GFP fluorescence was measured as IntDen and, since the data were obtained from several independent experiments, they were normalized by dividing by the geometric mean of the IntDen in the DMSO control group, thus expressing results as Log Fold inductions.

In the in vitro assays with U251-MG cell cultures, data normalization was performed by dividing by the geometric mean of the corresponding solvent control group. The mixture dose-response experiments were reproduced twice in vivo and in the ERα assay, and three times in the ERβ2 assay. However, due to high variability in replicate measures within each experiment, the three replicate datapoints at each concentration within each experiment were averaged, and the mixture dose-response model was based on two (ERα) or three (ERβ2) average values at each concentration. Moreover, in the ERβ2 assay, the maximum responses varied between experiments, and the data were therefore expressed as a percent of log-fold induction produced by E2 in each experiment.

### 4.7. Concentration-Response Modeling

The relationship between concentration and log-fold induction was modeled with a 4-parameter Hill model:

(1) $$\Phi \left(c\right)=Min+\frac{Max-Min}{1+{\left(\frac{c}{EC50}\right)}^{\beta }}$$

where *Min* is the minimum level of induction, *Max* is the maximum level of induction, *c* is the concentration, *EC*50 is the concentration producing 50% of the maximum induction, and *β* is the Hill slope. The first step of the dose-response analysis in both in vivo and in vitro experiments was to estimate common values of *Min* and *Max* for both single compounds within each biological model. The models with a common *Min* and *Max* on the one hand and with freely varying *Min* and *Max* on the other hand were compared with lack-of-fit *F*-tests. Appropriateness of the dose-response models were also tested with a test for lack-of-fit compared to ANOVA models. As a second step, common values of *Min*, *Max* and *Slope* were estimated for E2 and GEN. Lack-of-fit *F*-tests were performed to check that the model did not fit less well than when the slope varied freely. The parameters were estimated by least squares, using R 3.1.1 [38] and package drc [39]. All the data and R codes used in this study are available in Supplementary materials (Supplementary Figure S3).

### 4.8. Mixture Experimental Designs

The mixture experimental design was developed based on relative potency of single compounds according to [12].

In the in vivo assay, relative potency of E2 and GEN were estimated with the EC50 obtained by modelling dose-response data from respectively four and two experiments with the 4-parameter Hill model. Mixture experiments were performed according to a ray design with one ray for each single chemical and three mixture ratios (3:1, 1:1, and 1:3 expressed as relative potencies). Each ray was tested with five concentrations with 4-fold serial dilutions, centered around the EC50, except for GEN for which the highest concentrations were reduced because of their toxicity to fish (range of E2 concentrations from 4.8 × 10^−12^ to 5 × 10^−9^ M and range of GEN concentrations from 2.4 × 10^−8^ to 6.25 × 10^−6^ M) (Table 1). In theory, interactions are likely to be most visible at the equimolar mixture ratios.

The same approach was used to design the experiments for the in vitro assay with U251-MG cells. To calibrate the design, we used one experiment with E2 and GEN performed on the same plate. The ray designs built for the in vitro assays are presented in Table 2 for both ERα and ERβ2.

### 4.9. Mixture Concentration-Response Modeling

The mixture concentration-response modeling was performed as described in [12] with some adjustments when necessary. Concentration-response surfaces were modeled with the CA model [19] under the assumption of absence of interactions, using Berenbaum’s general solution [40]. This application of the CA model can be used in cases where the dose-responses of the mixture components produce same minimal and maximal effect: it does not require equal slopes in the dose-response models for single compounds. The use of the CA model when slopes differ remains a subject of controversy, because this would suggest that the single compound’s modes of action are different [41,42]. In agreement with Berenbaum’s general solution, the concept of Toxic Equivalent Factors, where slopes are required to be equal, is viewed as a more restrictive version of CA [43]. Prior to modelling the dose-response surface, individual rays were modelled with a Hill model with either freely varying slopes and EC50s or simply freely varying EC50s when this was not detrimental to the goodness-of-fit. On the other hand, Faust et al. [44] underline that their results do not support the idea that CA can only be applied with similar DR curves. Other authors believe that differences in Hill parameters or even differences in dose-response functions do not necessarily imply different sites of action and consider that the heterogeneity of binding sites can imply more complex dose-response functions [45]. CA has however provided adequate predictions even for mixtures where the mode of action was not identical [46].

A variety of methods have been developed for quantifying interactions based on analysis of specifically designed mixture dose-response data [47]. These include graphical methods that quantify deviations from isoboles [48,49], the widely-used Combination Index designed by Chou and Talalay [50,51], statistical methods for testing local departure from additivity [52], and modelling of the entire dose-response surface [47]. The method developed by [52] and dose-response surface modeling both allow appropriate error structure modelling [53,54] statistical tests of the significance of departures from the no-interaction model. Nonlinear response-surface analysis has the additional advantage of allowing for more complex interactions that depend on the response level or the mixture ratio [18] which could be relevant especially for endocrine disrupting compounds.

Interaction terms for simple antagonism/synergy (SA), dose-ratio dependent interactions (DR), and dose-level dependent interactions (DL) were subsequently added to the CA model [18]. These interaction models developed by Jonker et al. (2005) [18] allow for different slopes for the single compounds, but the interactions can be either calculated on toxic units based on the EC50 or on the EC at the response level under study:

$$z_{i}=\frac{TUx_{i}}{\sum_{j=1}^{n}TUx_{j}}$$

where either $TUx_{i}=\frac{c_{i}}{EC50_{i}}$ or $TUx_{i}=\frac{c_{i}}{ECx_{i}}$ For example, for simple synergism or antagonism, these toxic units are used in the following deviation function used by Jonker et al. (2005) [18].

$$G(z_{1},\ldots ,z_{n})=a\prod_{i=1}^{n}z_{i}$$

We therefore tested both implementations of the interaction definitions. Significance of the interactions was assessed using approximate *F*-tests on the residual sums of squares by considering that the models were nested. Acceptability of the concentration-response surface models was assessed with a lack-of-fit *F*-test compared to the analysis of variance model. Optimisation of parameter values for the dose-response surfaces was performed with R 3.1.1 [38], package dfoptim [55].

## 5. Conclusions

In the present study, we confirm (i) that our in vitro (U251-MG cells) and in vivo (*cyp19a1b*-GFP transgenic zebrafish) biological models are valuable tools to assess the estrogenic potency of chemicals both alone and in mixtures as previously stated [12]; (ii) the usefulness of the ray design approach to highlight interactions between mixture components in providing surface dose-response data and simple graphical representations. Our results show that mixture of two ER agonists, a phytoestrogen (GEN) and a natural estrogen (E2), could produce effects that deviate from the assumption of simple additivity, i.e., antagonistic effects, demonstrating the importance of considering chemical mixtures for a better understanding of the effects of ER agonists on organisms. From that point of view, both the U251-MG transactivation assay and the *cyp19a1b*-GFP transgenic zebrafish assay are reliable, flexible and simple assays, useful for the complex experimental design needed for mixture testing. Although extrapolation from the present assays to environmental situations appears difficult, as reported in our previous study [12], this assay could possibly help in determining the interactions of compounds in multi-component mixtures and/or identify compounds/mixtures that need further investigations in in vivo studies with more integrative endpoints.

## Figures and Tables

**Table 1:** Experimental ray design for the assessment of the effects of estradiol (E2) and genistein (GEN) alone and in mixtures on the expression of GFP in the brain of *cyp19a1b*-GFP transgenic zebrafish line.

Condition	[E2] (M)	[GEN] (M)	Ray
1	0	0	-
2	5.00 × 10^−9^	0	1:0
3	1.25 × 10^−9^	0	1:0
4	3.12 × 10^−10^	0	1:0
5	7.81 × 10^−11^	0	1:0
6	1.95 × 10^−11^	0	1:0
7	3.75 × 10^−9^	6.25 × 10^−6^	3:1
8	9.37 × 10^−10^	1.56 × 10^−6^	3:1
9	2.34 × 10^−10^	3.91 × 10^−7^	3:1
10	5.86 × 10^−11^	9.77 × 10^−8^	3:1
11	1.46 × 10^−11^	2.44 × 10^−8^	3:1
12	1.25 × 10^−9^	6.25 × 10^−6^	1:1
13	6.25 × 10^−10^	3.12 × 10^−6^	1:1
14	1.56 × 10^−10^	7.81 × 10^−7^	1:1
15	3.91 × 10^−11^	1.95 × 10^−7^	1:1
16	9.77 × 10^−12^	4.88 × 10^−8^	1:1
17	3.12 × 10^−10^	4.69 × 10^−6^	1:3
18	1.56 × 10^−10^	2.34 × 10^−6^	1:3
19	7.81 × 10^−11^	1.17 × 10^−6^	1:3
20	1.95 × 10^−11^	2.93 × 10^−7^	1:3
21	4.88 × 10^−12^	7.32 × 10^−8^	1:3
22	0	6.25 × 10^−6^	0:1
23	0	3.12 × 10^−6^	0:1
24	0	1.56 × 10^−6^	0:1
25	0	3.91 × 10^−7^	0:1
26	0	9.77 × 10^−8^	0:1

**Table 2:** Experimental ray design for the assessment of the effects of estradiol (E2) and genistein (GEN) alone and in mixtures on the luciferase activity in U251-MG cells transfected with zebrafish ERs.

	ERα		ERβ2		
Condition	[E2] (M)	[GEN] (M)	[E2] (M)	[GEN] (M)	Ray
1	0	0	0	0	-
2	2.00 × 10^−8^	0	2.00 × 10^−8^	0	1:0
3	2.00 × 10^−9^	0	2.00 × 10^−9^	0	1:0
4	2.00 × 10^−10^	0	2.00 × 10^−10^	0	1:0
5	2.00 × 10^−11^	0	2.00 × 10^−11^	0	1:0
6	2.00 × 10^−12^	0	2.00 × 10^−12^	0	1:0
7	1.50 × 10^−8^	1.00 × 10^−6^	1.50 × 10^−8^	2.00 × 10^−7^	3:1
8	1.50 × 10^−9^	1.00 × 10^−7^	1.50 × 10^−9^	2.00 × 10^−8^	3:1
9	1.50 × 10^−10^	1.00 × 10^−8^	1.50 × 10^−10^	2.00 × 10^−9^	3:1
10	1.50 × 10^−11^	1.00 × 10^−9^	1.50 × 10^−11^	2.00 × 10^−10^	3:1
11	1.50 × 10^−12^	1.00 × 10^−10^	1.50 × 10^−12^	2.00 × 10^−11^	3:1
12	1.00 × 10^−8^	2.00 × 10^−6^	1.00 × 10^−8^	4.00 × 10^−7^	1:1
13	1.00 × 10^−9^	2.00 × 10^−7^	1.00 × 10^−9^	4.00 × 10^−8^	1:1
14	1.00 × 10^−10^	2.00 × 10^−8^	1.00 × 10^−10^	4.00 × 10^−9^	1:1
15	1.00 × 10^−11^	2.00 × 10^−9^	1.00 × 10^−11^	4.00 × 10^−10^	1:1
16	1.00 × 10^−12^	2.00 × 10^−10^	1.00 × 10^−12^	4.00 × 10^−11^	1:1
17	5.00 × 10^−9^	3.00 × 10^−6^	5.00 × 10^−9^	6.00 × 10^−7^	1:3
18	5.00 × 10^−10^	3.00 × 10^−7^	5.00 × 10^−10^	6.00 × 10^−8^	1:3
19	5.00 × 10^−11^	3.00 × 10^−8^	5.00 × 10^−11^	6.00 × 10^−9^	1:3
20	5.00 × 10^−12^	3.00 × 10^−9^	5.00 × 10^−12^	6.00 × 10^−10^	1:3
21	5.00 × 10^−13^	3.00 × 10^−10^	5.00 × 10^−13^	6.00 × 10^−11^	1:3
22	0	4.00 × 10^−6^	0	8.00 × 10^−7^	0:1
23	0	4.00 × 10^−7^	0	8.00 × 10^−8^	0:1
24	0	4.00 × 10^−8^	0	8.00 × 10^−9^	0:1
25	0	4.00 × 10^−9^	0	8.00 × 10^−10^	0:1
26	0	4.00 × 10^−10^	0	8.00 × 10^−11^	0:1

## Supplementary Materials

Supplementary materials can be found at http://www.mdpi.com/1422-0067/19/4/1047/s1.

## Abbreviations

CA	Concentration Addition
DL	Dose-Level dependent interaction model
E2	17β-estradiol
EC50	Effective Concentration 50%
ER	Estrogen Receptor
FCS	Fetal Calf Serum
GEN	Genistein
GFP	Green Fluorescent Protein
SA	Simple Interaction model
