# Supplementary material for: Mixture Concentration-Response Modeling Reveals Antagonistic Effects of Estradiol and Genistein in Combination on Brain Aromatase Gene (cyp19a1b) in Zebrafish
Source: Int J Mol Sci. 2018 Apr 1;19(4):1047. doi: 10.3390/ijms19041047 (PMC5979603; doi:10.3390/ijms19041047)
Supplement: Supplementary file 1 [file ijms-19-01047-s001.zip › Supplementary Figure 1 and 2.pdf]

Supplementary Materials: Mixture Concentration-Response Modeling Reveals Antagonistic Effects of Estradiol and Genistein in Combination on Brain Aromatase Gene (*cyp19a1b*) in Zebrafish

Hinfray Nathalie, Tebby Cleo, Piccini Benjamin, Bourgine Gaelle, Aït-Aïssa Sélim, Porcher Jean-Marc, Pakdel Farzad and Brion François

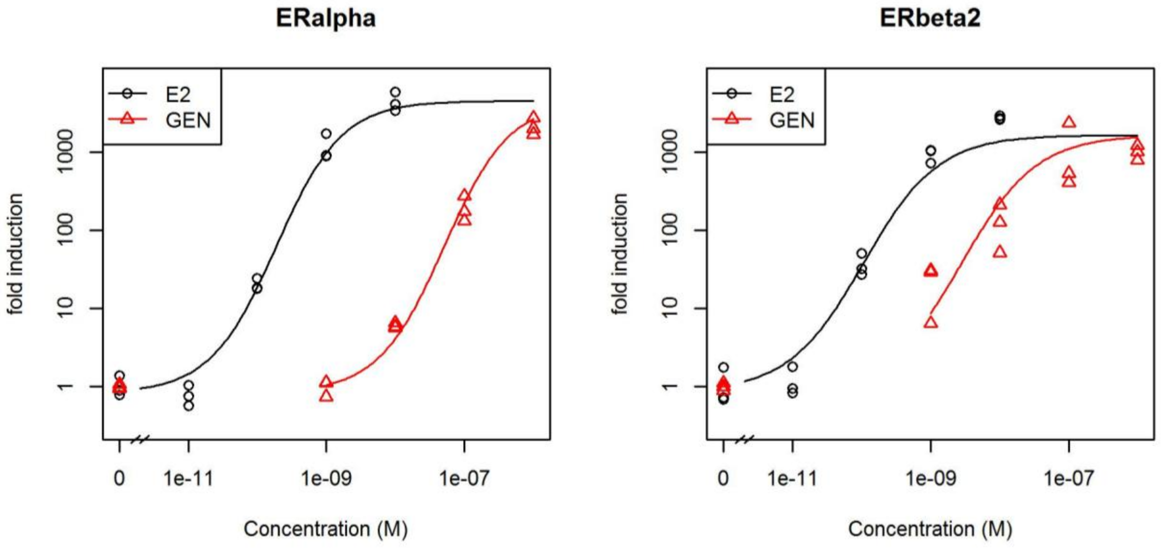

Figure S1. Concentration-response curves of luciferase activity in U251-MG cells transfected with ERalpha or ERbeta2 after exposure to estradiol (E2) or genistein (GEN). These data originated from one concentration-response experiment for each compound and each ER with 3 replicated wells for each condition.

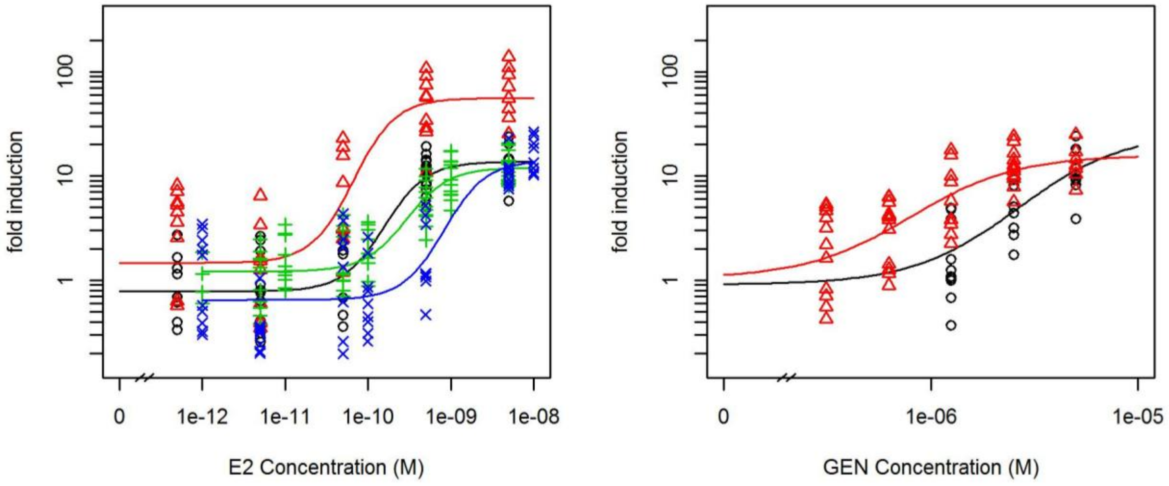

Figure S2. Concentration-response curves of GFP fluorescence *in vivo* in the brain of 4-dpf old *cyp19a1b*-GFP transgenic zebrafish after exposure to estradiol (E2) or genistein (GEN) for 96 hours. These data originated from 2 (GEN) to 4 (E2) independent experiments with 8–19 transgenic zebrafish per condition.
